# Supplementary material for: Spite is contagious in dynamic networks
Source: Nat Commun. 2021 Jan 11;12:260. doi: 10.1038/s41467-020-20436-1 (PMC7801472; doi:10.1038/s41467-020-20436-1)
Supplement: Supplementary file 1 — Supplementary Information [file 41467_2020_20436_MOESM1_ESM.pdf]

Supplementary Materials for:
Spite is contagious in dynamic networks

Zachary Fulker[1], **Patrick Forber**[2], **Rory Smead**[3], **and Christoph Riedl**[1,4,5,6,*]

[1]Network Science Institute, Northeastern University, Boston, MA
[2]Department of Philosophy, Tufts University, Medford, MA
[3]Department of Philosophy and Religion, Northeastern University, Boston, MA
[4]D'Amore-McKim School of Business, Northeastern University, Boston, MA
[5]Khoury College of Computer Sciences, Northeastern University, Boston, MA
[6]IMT Lucca, Piazza S. Ponziano, 6, 55100 Lucca, Italy
[*]c.riedl@neu.edu

nature COMMUNICATIONS

Contents

Supplementary Note 1: The Classic Anti-Correlated Interaction Threshold for Spite 2
Supplementary Note 2: Measure of Endogenous Correlated Interaction 2
Supplementary Note 3: Importance of Coevolution of Strategy and Network 3
Supplementary Note 4: Comparison to Prisoner's Dilemma 3
Supplementary Note 5: Robustness Tests 4
Supplementary Note 6: A Biological Model 6
References 8

Supplementary Note 1: The Classic Anti-Correlated Interaction Threshold for Spite

Previous studies[1–3] established a general condition for the evolution of spite in infinite populations with a uniform rate of anti-correlation (also called "negative assortment"). Assume an infinite population of individuals playing the Prisoner's Delight. Let x be the frequency of 'Social' types, $(1-x)$ be the frequency of 'Spiteful' types, r be the proportion of anti-correlated interactions—the probability that a given type interacts with an agent of the opposite type—and $(1-r)$ the proportion of random interactions. Note that this approach to non-random interactions has also been used when studying the evolution of cooperation[4,5]. Other studies often use the word "assortment" rather than "correlated interactions" and this exogenous r-parameter is intended to represent non-random pairing of individuals in the population without specifying a particular mechanism by which the pairing occurs. Using this framework, the fitness of each strategy is then:

$$F(Social,x) = (1-r)(x+(1-x)c)+rc, \tag{1}$$

$$F(Spiteful,x) = (1-r)bx+rb. \tag{2}$$

'Spiteful' will be favored over 'Social' whenever $F(Spiteful,x) > F(Social,x)$ or:

$$(1-r)bx+rb > (1-r)(x+(1-x)c)+rc. \tag{3}$$

Which reduces to:

$$r > c/b. \tag{4}$$

This approach to understanding spiteful behavior is limited in some important ways. First, note that if an individual changes strategies, it is assumed that their interaction partners change immediately. This is neither realistic, nor does it fit with traditional game-theoretic assumptions of independent strategy choice by each player. Second, because the mechanism causing the correlated interactions (assortment) is unspecified it is difficult to assess the relevance of these abstract models to particular phenomena. It is for these reasons that our model does not use an exogenous correlated interaction (assortment) parameter, but rather we model partner choice as an endogenous response to payoffs received in previous interactions and reference the classic $r > c/b$ inequality only for comparison with our results.

Supplementary Note 2: Measure of Endogenous Correlated Interaction

In our dynamic network model, we measure the degree of endogenous correlated interactions as how much interaction patterns diverge from a uniformly mixing population. A correlated interaction value of a measures the frequency of interactions an agent has with others of a specific strategy type relative to the expectation for random mixing. If a is positive then agents are interacting with others of the same type more than expected under random mixing. If a is negative then agents are interacting with opposite types more than expected under random mixing. Agents with positive a are engaged in *correlated* interactions, whereas agents with negative a are engaged in anti-correlated interactions. For example, if a 'Spiteful' agent is expected to have 0.4 of their interactions with other 'Spiteful' agents, but the proportion of 'Spiteful' agents in the population is 0.5, then that agent has a measure of (anti-)correlated interaction of -0.1.

The measure a_i for an individual i is calculated as specified in the Methods section. We can also calculate the measure for a given type in the population by averaging the a_i values for all i of a given strategy. Denote these as: a_{social} and a_{spite}, respectively. Finally, let $\bar{a}$ be the average measure for the whole population. Note that on this measure it is possible, as in our case, that different types of agents form opposing correlation patterns that when averaged offset. Because of this, the overall degree of correlated interactions may obfuscate meaningful interaction patterns at the level of the individual and strategy type.

The classic threshold is derived form assuming a uniform amount of correlated interaction across the population (all individuals experience the same degree of correlated or anti-correlated interaction), which is not true in dynamic networks. However, in a population with uniform anti-correlated interaction, it is is possible to derive a direct connection between our measure and the classic representation. Suppose there is a large population that has a degree of anti-correlated interaction of r, and that this population is otherwise uniform in structure. The a-values for each type in this population can be calculated as:

$$a_{Social} = (1-r)x - x, \tag{5}$$

$$a_{Spiteful} = (1-r)(1-x) - (1-x). \tag{6}$$

And the aggregate measure for the whole population is:

$$\bar{a} = x a_{Social} + (1-x) a_{Spiteful}. \tag{7}$$

Which reduces to:

$$\bar{a} = -r(1-2x+2x^2). \tag{8}$$

Note that the relation between $\bar{a}$ and r depends on the specific population frequencies. However, because $x \in [0,1]$, the factor $(1-2x+2x^2)$ will always fall in the interval $[0.5,1]$ and hence $\bar{a} \in [-r, -r/2]$ for any value of x. Thus, the classic $r > c/b$ threshold, translated into an expected a-value that falls in the interval $[-c/b, -c/2b]$ depending on the value of x, where $\bar{a} \to -c/b$ as $x \to 1$ or $x \to 0$, and $\bar{a} \to -c/2b$ as $x \to 0.5$. When determining either stability or invasion conditions for spite in traditional infinite population models one should examine $x \approx 0$ or $x \approx 1$ respectively and, in either case, the relevant baseline for the traditional measure is $\bar{a} \approx -c/b$. It is for this reason that we use $-c/b$ as the baseline for comparison when presenting our results.

Incidentally, the measure a (see Methods) is formally analogous to the Price equation definition of relatedness coefficients as deviations between the allele frequencies in groups of actors/recipients and the population average allele frequencies[6,7].

Supplementary Note 3: Importance of Coevolution of Strategy and Network

The endogenous partner choice in our model is essential for the spread of spite. To demonstrate this, consider an example on a fixed network. Building on our analytic model, consider a network such that each agent has one incoming and one outgoing link, they cannot update these links, and the links form a ring as depicted below.

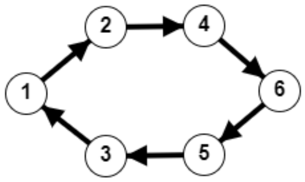

Now suppose agent 1 is 'Spiteful' and the rest are 'Social'. At the first time step it follows that agents 2 and 3 will imitate the 'Spiteful' strategy if $2b-c > 1$. At time-step 2, however, agents 4 and 5 will not imitate the 'Spiteful' strategy of agents 2 and 3 because $U_2^2 = U_3^2 = b/2$ is less than $U_4^2 = U_5^2 = (c+1)/2$. 'Spiteful' agents are no longer disproportionately interacting with only 'Social' agents. Consequently, on the next time-step, 'Spiteful' agents 2 and 3 will imitate the 'Social' strategy of 4 and 5 and the system is back to its initial state. In all future rounds agents 2 and 3 will continue to alternate between 'Spiteful' and 'Social'. This shows that there are static networks on which spite is unable to spread. This simple example illustrates that imitation alone is not sufficient for the spiteful behavior to spread throughout the entire population, and a dynamic network (i.e., partner choice) is essential. Only with the ability to shed connections to other 'Spiteful' agents, especially those that recently changed strategies, can 'Spiteful' agents persistently target 'Social' agents and enable the spread of spite.

Supplementary Note 4: Comparison to Prisoner's Dilemma

We have used the Prisoner's Delight to explore spiteful behavior. The more famous Prisoner's Dilemma (see Table S1) has been used to study altruistic and cooperative behavior. The relationship between altruism and spite, and consequently the Delight and Dilemma, is somewhat complicated. Both spite and altruism represent dominated strategies and so are similar in many evolutionary dynamics (e.g., the replicator dynamics). However, interaction partners invariably prefer to interact with altruists (cooperators in the Dilemma) and never prefer spiteful types. There are also some formal similarities in the classic conditions necessary for the evolution of altruism (Hamilton's Rule) and spite. These factors have led some scholars to argue that spite is a form of indirect altruism[8]. Others argue that apparent spite is usually a form of selfishness[2]. And others argue that it is distinct from both selfishness and altruism[3]. While our results here do not settle these issues, it is worthwhile to compare our results on spite in the Prisoner's Delight with those of the Prisoner's Dilemma. Results from our model reveal that, while there are some parallels, there are also important differences between the two games.

Figure S1 compares the results of our model (which uses the Prisoner's Delight) with a version using the classic Prisoner's Dilemma. Using the Dilemma, our results mirror those of previous studies of that game[9,10]. The comparison between the two games is noteworthy in several ways. First, the general network dynamic is similar: agents playing 'Cooperate' preferentially interact with one another and avoid agents playing 'Defect', just as 'Social' agents avoid 'Spiteful' agents in the Delight. Second, the resulting strategic evolution is dependent on the kind of imitation rule used. If agents consider total payoff when

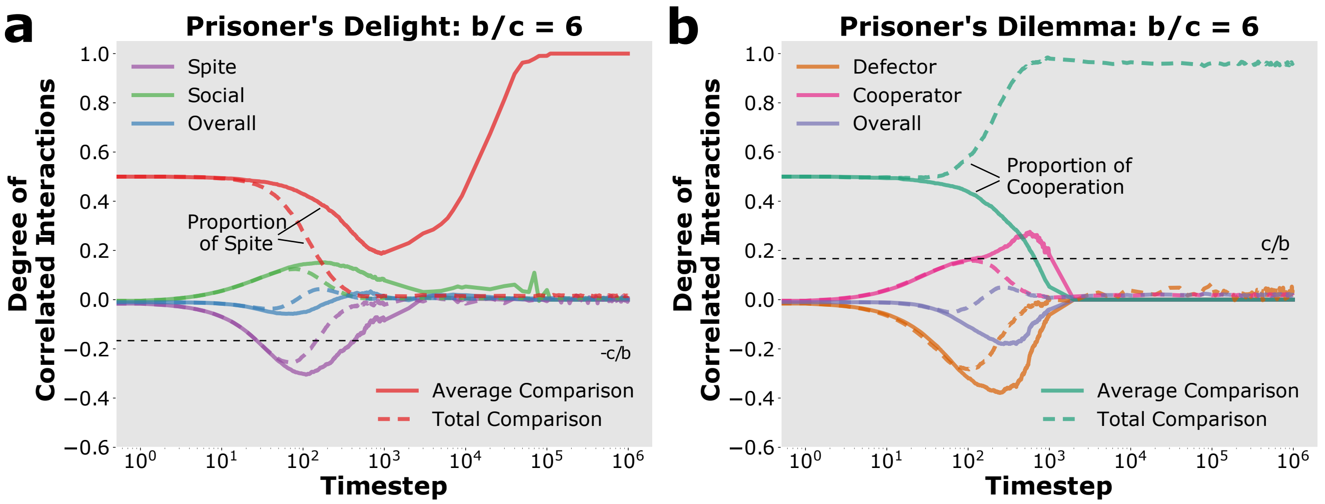

Figure S1. Comparison between the Prisoner's Delight and the Prisoner's Dilemma. (a) Results for the Prisoner's Delight showing the proportion of correlated interactions between strategies and evolution over time. 'Spiteful' becomes negatively correlated and evolves when agents imitate based on average payoffs. **(b)** Results for the Prisoner's Delight showing the proportion of correlated interactions between strategies and evolution over time. 'Cooperate' becomes positively correlated and evolves when agents imitate based on total payoffs. For the Prisoner's Dilemma, the relevant baseline correlation measure for the stability and invasion conditions for 'Cooperate' is c/b rather than $-c/b$ for 'Spiteful' in the Delight.

imitating, evolution favors the 'Social' and 'Cooperate' strategies in the Delight and Dilemma, respectively. On the other hand, if agents consider average payoff then imitation will lead to the 'Spiteful'/'Defect' strategies. Third, in both cases the dynamic networks show there are scenarios where dominated strategies ('Spiteful' in the Delight and 'Cooperate' in the Dilemma) will spread, although the specifics, and the nature of the imitation dynamic in particular, differ in each case. Thus, the same mechanism (dynamic networks) can produce either altruism or spite depending on how imitation occurs. The reason for this difference is that spite spreads by generating relative advantages, at an absolute cost, on a per-interaction basis. If agents track total payoff rather than average payoff, they become less sensitive to these relative advantages and imitation become more sensitive to the total number of interactions. Both agents playing 'Cooperate' in the Dilemma and 'Social' agents in the Delight attract more visitors and have more interactions, and consequently have higher total payoffs.

	Cooperate	Defect
Cooperate	b, b	0, b+c
Defect	b+c, 0	c, c

Table S1. The Prisoner's Dilemma. Traditionally 'Cooperate' agents pay a cost c to confer a benefit b on their partners. The matrix here is an affine transformation of the traditional matrix normalized so all payoffs are non-negative. As with the Delight, we assume $b+c=1$.

Supplementary Note 5: Robustness Tests

Starting Proportion

Most of the simulations we ran started with an equal proportion of 'Spiteful' and 'Social' agents. To investigate if the emergence of spite was robust to only a single initially 'Spiteful' agent we ran several more simulations. We find our results are robust and that the invasion of spite is possible even when there is only one 'Spiteful' agent (Figure S2). Note that in many of these cases, spite would go extinct only to be re-introduced through an error in imitation before then spreading through the entire population. Regardless, however with $b/c = 6$ every simulation had converged to 100% 'Spiteful' agents well before the 10^6 time-step.

Network Learning Speed

In order to demonstrate the effect of network learning speed on the interaction patterns in our model over time, a slower network learning speed (0.1) and faster learning speed (10) are compared. A faster network learning speed leads to a greater magnitude

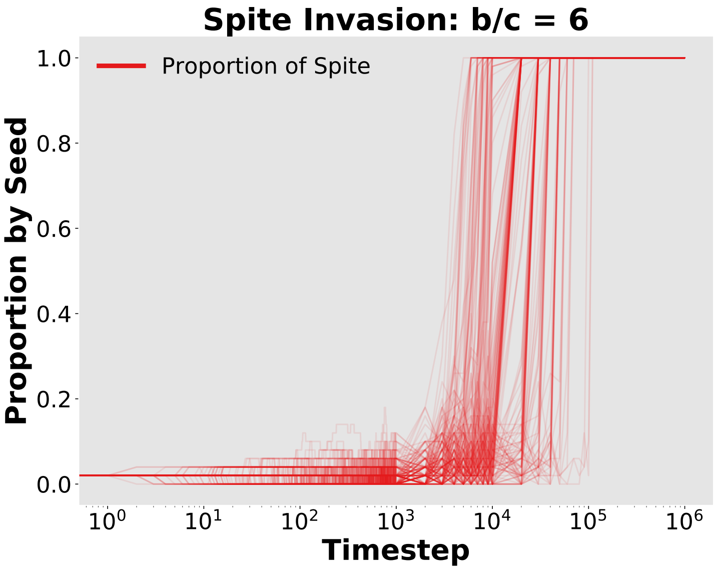

Figure S2. The emergence of spite can occur when there is only a single 'Spiteful' agent. This demonstrates how spite could invade a population from a single mutated agent.

of correlated behaviors and a quicker adoption of spiteful behaviors, but the learning speed does not alter the final outcome given a sufficient number of time-steps (Figure S3). Note that there is an important difference between these results and those with varying the learning discount rate (Figure 3) that warrants explanation. Recall the updating rule for link weights:

$$w'_{ij} = (1-\delta)w_{ij} + R\pi_i. \tag{9}$$

Given that both R and $(1-\delta)$ raise link weights, one may expect that a high R and a low δ will have similar effects. However, note that $(1-\delta)$ is applied to total link weights at a given round, meaning that the effect of δ is the same on every round, despite the fact that w_{ij} is significantly increasing during the course of the simulation. Consequently, a low δ allows accumulated weights to become very difficult to change. On the other hand, for the values of R we examine here, the accumulating weights quickly overwhelm any impact of a multiplier on the payoffs because the payoff function π remains constant over time. Thus R has a significant impact on the initial speed of learning, but does not impact the resulting final states as seen with lower δ values.

Payoff Structure

For our main results it was assumed that $b+c=1$. This allowed us to focus on the relevance of the b/c ratio. In order to test if fixing $b+c=1$ influenced model results, we ran simulations with $b+c<1$ while still keeping the 'Social'-'Social' payoff fixed at 1 (see payoffs in Table S2). In addition to robustness testing, this modification allows us to explore the possibility that the payoffs are not additive and that there may be some synergistic benefits of the 'Social' strategy. We find that $b+c$ can be less than 1 and spite will still emerge (Figure S4). If the $b+c$ value is too small, however, the cost of spite becomes too large and the 'Social' strategy emerges. In line with the rest of our results, the larger the b/c ratio, the more spite is favored and the smaller the value of $b+c$ can be while still producing spite.

	Spite	Social
Spite	0, 0	b, c
Social	c, b	1, 1

Table S2. The Prisoner's Delight with non-additive payoffs. For our main results the 'Social'-'Social' payoff was equivalent to $b+c(=1)$. Here we examine model outcomes with the 'Social'-'Social' payoff fixed to 1, but $b+c<1$.

Additionally, when $b+c=1$ it can be shown that 'Spiteful' agents learn to target 'Social' agents relatively faster than

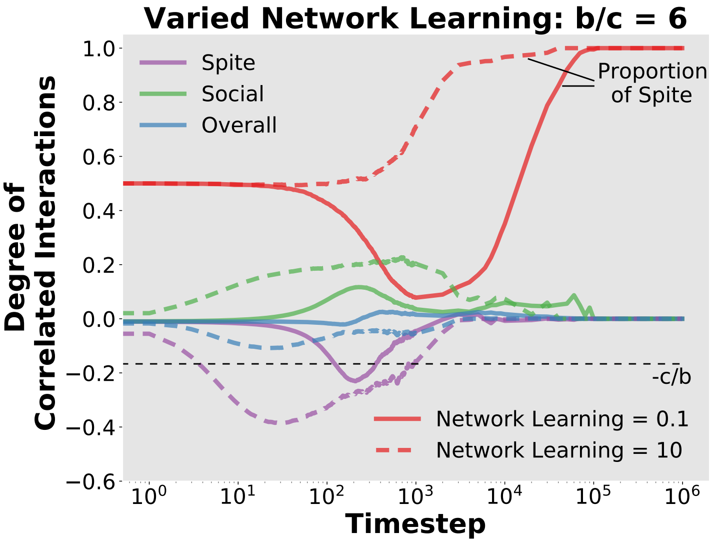

Figure S3. The network learning speed effects the speed and magnitude of the formation of (anti)correlation. However, over a wide range of learning speeds spiteful behavior becomes the norm.

’Social’ agents learn to target ’Social’ agents as a simple function of reinforcement learning.

$$\frac{w+b}{w} > \frac{w+1}{w+c} = \frac{w+b+c}{w+c} \tag{10}$$

Where w is some constant. Our simulation results show this is not a necessary condition for the emergence of spite. For example, Figure S4a shows spite emerges when $b+c=0.95$ with $b=0.81428$, $c=0.13571$, and $b/c=6$. This is made clear in the inequality

$$\frac{w+0.81428}{w} < \frac{w+1}{w+0.13571}. \tag{11}$$

This demonstrates spite can emerge even when ’Social’ agents learn to target ’Social’ agents relatively faster than ’Spiteful’ agents target ’Social’ agents.

Supplementary Note 6: A Biological Model

In order to address a more biological interpretation of our results we implemented a version of our model using a version of the frequency dependent Moran Process[11,12] in place of imitation. Under these model settings an agent was randomly selected to die every 10 rounds. The dying agent is replaced by a new agent with reinitialized network weights and given a new strategy type selected in proportion to the success of each strategy type over the previous 10 rounds. Specifically, the probability a new agent adopts strategy s is:

$$Pr(s) = N_s\left(\frac{\bar{P}_s}{N*\bar{P}}\right)$$

Where N_s is the number of agents of strategy type s, $\bar{P}_s$ is the average payoff per interaction of strategy type s, N is the total number of agents, and $\bar{P}$ is the average payoff received across all interactions. The Moran process results demonstrate the same general trends seen in the imitation learning model (Figure S5).

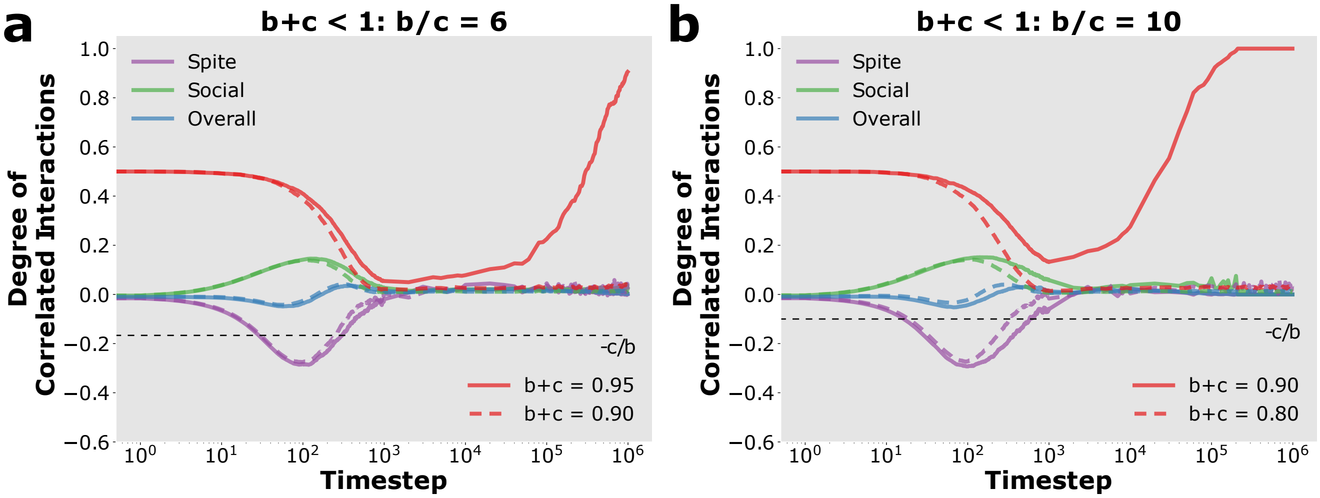

Figure S4. **The emergence of spite is robust to breaking the assumption $\mathbf{b+c=1}$. (a)** The value of $b+c$ can be less than 1 and still support the emergence of spite. If the value of $b+c$ is too small, however, the cost of spite is too high and the social strategy emerges. **(b)** The larger the b/c ratio, the smaller the value of $b+c$ can be while still getting an emergence of spite.

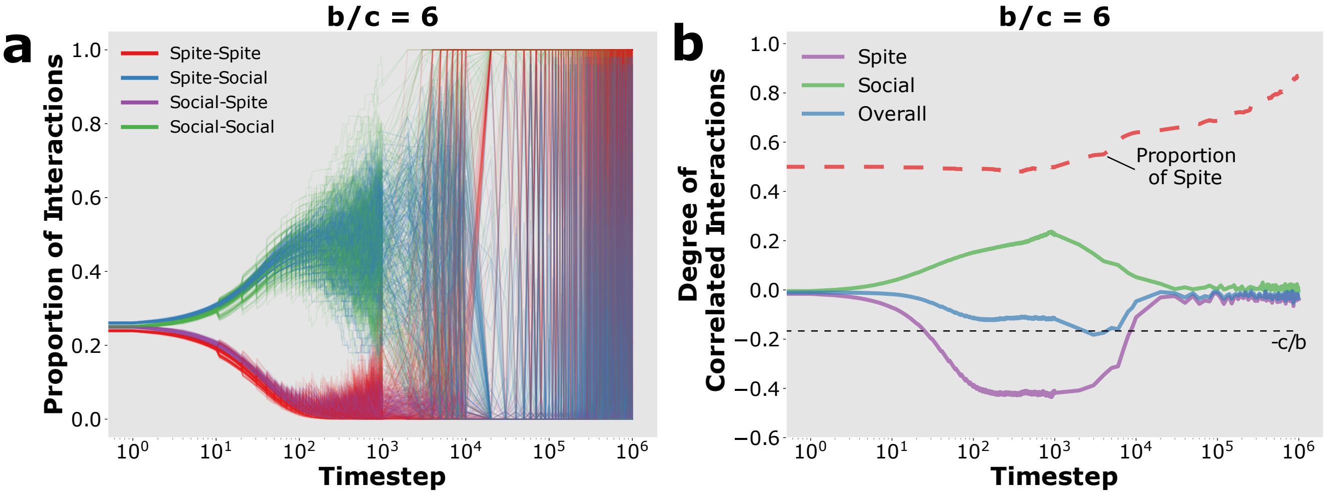

Figure S5. **(a)** As expected the Moran Process results contain more noise but the same general pattern of evolving interaction probabilities arises. 'Spiteful' agents learn to target 'Social' agents, and 'Social' agents learn to target each other. **(b)** Again the system level outcome of the changing composition of interactions produces negative assortment for 'Spiteful' agents and positive assortment for 'Social' agents. The simultaneous emergence of both assortment types leads to an increase in spiteful behavior.

References

1. Hamilton, W. Selfish and spiteful behaviour in an evolutionary model. *Nature* **228**, 1218–1220 (1970).
2. West, S. A. & Gardner, A. Altruism, spite, and greenbeards. *Science* **327**, 1341–1344 (2010).
3. Smead, R. & Forber, P. The evolutionary dynamics of spite in finite populations. *Evolution* **67**, 698–707 (2012).
4. Eshel, I. & Cavalli-Sforza, L. L. Assortment of encounters and evolution of cooperativeness. *Proc. Natl. Acad. Sci.* **79**, 1331–1335 (1982).
5. Skyrms, B. *Evolution of the social contract* (Cambridge University Press, 1996).
6. Grafen, A. A geometric view of relatedness. *Oxf. surveys evolutionary biology* **2**, 28–89 (1985).
7. Madgwick, P. G. Spite and the geometry of negative relatedness. *The Am. Nat.* doi.org/10.1086/710764 (2020).
8. Lehmann, L., Feldman, M. W. & Rousset, F. On the evolution of harming and recognition in finite panmictic and infinite structured populations. *Evol. Int. J. Org. Evol.* **63**, 2896–2913 (2009).
9. Skyrms, B. & Pemantle, R. A dynamic model of social network formation. *Proc. Natl. Acad. Sci.* **97**, 16 (2000).
10. Skyrms, B. *The stag hunt and the evolution of social structure* (Cambridge University Press, 2004).
11. Moran, P. A. P. *et al.* The statistical processes of evolutionary theory. *Am. J. Hum. Genet.* **14**, 438—439 (1962).
12. Taylor, C., Fudenberg, D., Sasaki, A. & Nowak, M. A. Evolutionary game dynamics in finite populations. *Bull. Math. Biol.* **66**, 1621–1644 (2004).
